# Effects of psychological intervention on empathy fatigue in nurses: A meta-analysis

**DOI:** 10.3389/fpubh.2022.952932

**Published:** 2022-10-14

**Authors:** Xiaojuan Chen, Mingdi Chen, Huang Zheng, Chaoyu Wang, Huimin Chen, Qinglan Wu, Huizhao Liao, Jinru Zhu, Junyan Lin, Xudong Ou, Zhihong Zou, Zhiwei Wang, Zhenzhen Zheng, Xianrui Zhuang, Riken Chen

**Affiliations:** ^1^The Second Affiliated Hospital of Guangdong Medical University, Zhanjiang, China; ^2^Medical College of Jiaying University, Meizhou, China; ^3^Taishan Hospital of Traditional Chinese Medicine, Jiangmen, China; ^4^Central People's Hospital of Zhanjiang, Zhanjiang, China; ^5^State Key Laboratory of Respiratory Disease, National Clinical Research Center for Respiratory Disease, Guangzhou Institute of Respiratory Health, The First Affiliated Hospital of Guangzhou Medical University, Guangzhou Medical University, Guangzhou, China

**Keywords:** psychological intervention, empathy fatigue, burnout, empathy satisfaction, nursing staff, randomized controlled trial, meta-analysis

## Abstract

**Objective:**

The purpose of this meta-analysis is to systematically assess the effects of psychological intervention on empathy fatigue among nursing staff.

**Method:**

Five electronic databases are searched separately from their establishment to April 8th, 2022. The research team independently performs paper selection, quality assessment, data extraction and analysis for all included studies. PRISMA guidelines are used to report this meta-analysis.

**Results:**

A total of seven randomized controlled trials (RCTs) covering 513 nursing staff are included. The meta-analysis results show that the empathy fatigue score (SMD = −0.22, 95% CI: −0.42~−0.02, *P* = 0.03) and burnout (SMD = −0.37, 95% CI: −0.56~−0.19, *P* < 0.001) are lower than the control group. The empathy satisfaction score of the psychological intervention group is higher than that of the control group (SMD = 0.45, 95% CI: 0.27–0.63, *P* < 0.001). The differences are statistically significant (*P* < 0.05). Subgroup analysis finds significant heterogeneity in the impact of different departments on psychological intervention at ≥6 weeks (*I*^2^ = 71%, *P* = 0.01) and <6 weeks (*I*^2^ = 0%, *P* = 0.75) (*P* = 0.05). Different departments also show significant heterogeneity in the effects of psychological intervention: ICU (*I*^2^ = 73%, *P* = 0.02), pediatric (*I*^2^ = 53%, *P* = 0.14) and other departments (*I*^2^ = 0%, *P* = 0.63). The differences are statistically significant (*P* = 0.0007). Besides, the results show that both mindfulness intervention (SMD = 0.50, 95% CI: 0.24–0.77, *P* = 0.0002) and other interventions (SMD = 0.41, 95% CI: 0.16–0.65, *P* = 0.001) are statistically significant difference in the level of empathy satisfaction between the psychological intervention group and the control group.

**Conclusion:**

Psychological intervention has a coordinated improvement effect on empathy fatigue, empathy satisfaction and burnout, and can also improve the quality of life of nursing staff.

## Introduction

Empathy fatigue, also known as “the cost of caring,” refers to a kind of occupational hazard suffered by helping people in the process of providing assistance services to the injured population. It is a psychological problem characterized by indirect exposure to traumatic events on the premise of providing empathy to others (1). In 2005, Stamm (2) proposed a three-dimensional structural model of empathy fatigue, and measured it through the Professional Quality of Life Scale (ProQOL), which is a common tool for evaluating empathy fatigue in medical groups, which has good reliability and validity. Compassion and empathy are the cornerstones of practicing humane care, which require nurses to feel and perceive from the perspective of patients, and understand and help patients to cope with all kinds of pressure and pain. Furthermore, compassion and empathy are the basis of humanized care. Nurses are supposed to recognize and understand patients from their perspective, and help them to deal with the pain of disease and life pressure as much as possible. However, with the extension of nurses' working years, and due to long-term and repeated exposure to patients' pain, they often suffer from empathy fatigue, and have difficulty in feeling patients' suffering. If not adjusted in time, this will certainly affect their mental health, induce job burnout and even reduce the quality and safety of their work (1). Nursing staff is a high risk group for empathy fatigue, as they make contact with patients directly, continuously, closely and extensively (3). Empathy fatigue not only has a serious impact on the physical and mental health of nursing staff, but it also has a negative impact on the level of work input, patient safety and quality of medical services. It can even lead to medical error or the loss of nursing talents, which is a common phenomenon across cultures and regions (4). If the symptoms of empathy fatigue persist, nurses may decide that leaving is the only solution, leading to a shortage of nursing staff. The World Health Organization predicts that the global shortage of nursing staff is projected to reach 7.6 million by 2030. At present, nursing staff are the shortest in supply in the health care system (5, 6), Psychological intervention is a relatively preferable method to solve the symptom of empathy fatigue (7–9), which mainly includes mindfulness-based stress reduction therapy, Balint groups, high-quality nursing service systems and stress reduction management of head nurses, as well as other stress reduction methods. However, there are great differences in the effects, forms and duration of empathy fatigue among nursing staff in different areas. Therefore, we used a meta-analysis of psychological intervention to objectively evaluate the impact of empathy fatigue on nursing staff so as to provide a scientific basis and references for intervention.

## Methods

### Literature search strategy

We performed a preliminary scoping search of the Cochrane Library and PubMed databases to determine appropriate key words. We then performed systematic retrieval based on the key words in five electronic databases from their establishment to April 8th, 2022. The five databases were Cochrane Library, PubMed, Web of Science, CINAHL and Embase. According to the PICOS principles, the topics were divided into empathy fatigue, psychological intervention, nurses and randomized controlled trial. (1) P (Participants) - English search terms for nurses: nurses, nursing staff, nursing personnel; (2) I (Intervention) - English search terms for psychosocial intervention: psychosocial intervention, psychosocial interventions, mindfulness therapy, mindfulness, Compassion Fatigue Resiliency Program; (3) O (Outcomes) - English search terms for empathy fatigue: empathy fatigue, vicarious trauma, secondary trauma, decondary traumatization, secondary traumatic stress, vicarious traumatization, Professional Quality of Life Scale (ProQOL), empathy satisfaction, burnout; (4) S (Study Design) - English search terms for randomized controlled trial: randomized controlled trial, controlled clinical trial, random allocation, randomized, placebo, randomly. The retrieval was carried out using a combination of medical subject headings (MeSH) and free words, and adjusted according to the characteristics of each database. Each search term was connected with the word “OR,” then the four sets of results were connected with the Boolean operator “AND” to search the relevant literature. And the time frame for the searches included all literature before April 8th, 2022.

### Eligibility criteria

The inclusion criteria were:

(i) Study design: randomized controlled trial; (ii) participants: registered nurses; (iii) the study used the outcome indicator ProQOL; (iv) the study involved intervention measures such as mindfulness therapy, compassion fatigue resiliency programs, emotional regulation training, etc.; (v) the language of the included literature was English.

The exclusion criteria were:

(i) Study design: not randomized controlled trial; (ii) participants: non-nursing staff; (iii) systematic review articles, magazine articles, case reports, low-quality articles and so on; (iv) full articles unavailable.

### Study selection

We used Note Express software to import and manage the search results. After removing duplicates, two reviewers independently assessed the studies by title and abstract, then reviewed the full texts. Any disagreement was settled through discussion with a third reviewer.

### Data extraction

After the final list of articles was settled, two searchers used datasheets (Microsoft Excel) to independently extract the data from each article. The data extraction characteristics included: author, year, country, department, sample size of experimental group and control group, intervention methods of experimental group and control group, intervention time, intervention frequency, outcome indicator ProQOL, empathy fatigue, empathy satisfaction and burnout.

### Statistical analysis

We used Review Manager 5.4.1 software to merge the statistics of our previously extracted data. The original data included in this study is continuous variables. The meta-analysis results were expressed by standardized mean difference (SMD) and 95% CI. The sizes of the I2 and *P*-values were used to examine heterogeneity between studies. If I2 < 50%, *P* > 0.1, it indicated that there was no statistical heterogeneity among the research results, so the fixed effects model was used for meta-analysis. Instead, if I2 ≥ 50%, *P* < 0.1, it suggested that there was statistical heterogeneity among the research results, so the random effects model was used. The sources of heterogeneity could be analyzed by subgroup analysis or sensitivity analysis. The difference was statistically significant (*P* < 0.05).

## Results

### Search results

**Figure 1:**
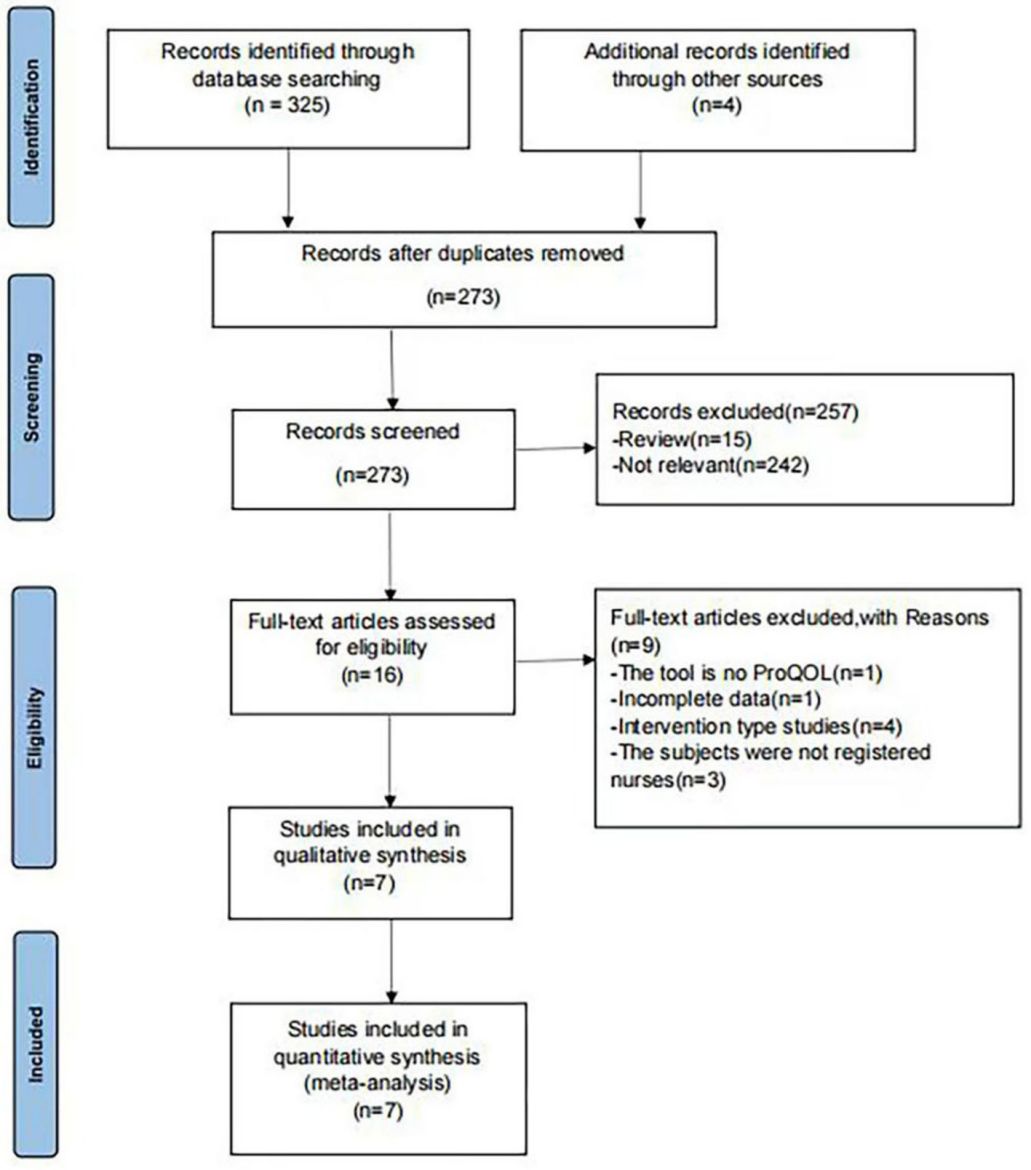
Flowchart of literature search.

The search was performed from March to May in 2022. A total of 329 articles were retrieved, of which 56 duplicate articles were excluded. Based on the inclusion and exclusion criteria, 257 articles were excluded by screening the titles and abstracts. The reasons for exclusion were: irrelevant (*n* = 247); reviews (*n* = 22). After reading the full texts, nine articles were excluded. Finally, a total of seven articles were included in the meta-analysis (see Figure 1) (7–13).

### Characteristics of included studies

In the seven articles covering 315 nurses from six different countries, most were from the ICU (*n* = 3), followed by the pediatric and other departments (Table 1).

**Table 1:** The characteristics of each study.

**Author, year**	**Country**	**Department**	**Sample** **(T/C)**	**Intervention**		**Time of** **intervention** **(weeks)**	**Frequency of intervention**
				**T**	**C**		
Berger and Gelkop (10)	Israel	Pediatric	42/38	Mindfulness therapy	Blank control	12	Twice a week, 90 min per week
Kang et al. (8)	Korean	ICU	15/23	Self-reflection program	Blank control	6	Once a week, 90 min per week
Wylde et al. (9)	USA	Pediatric	46/49	Mindfulness intervention	Regular discussion education	4	Once a week, 120 min per week
Slatyer et al. (7)	Australia	Neurosurgery	60/16	Mindfulness therapy	Blank control	7	Once a week, 105 min per week
Kharatzadeh et al. (11)	Iran	ICU	26/27	ERT	Blank control	6	Once a week, 120 min per week
Pehlivan and Güner (12)	Turkey	Oncology–hematology	49/42	Compassion Fatigue Resiliency Program	Blank control	5	Once a week, 120 min per week
Emani et al. (13)	Iran	ICU	40/40	Chromotherapy-based interventions	Blank control	5	Once a week, 120 min per week

T, treatment group; C, control group; ERT, emotional regulation training.

### Quality appraisal

**Figure 2:**
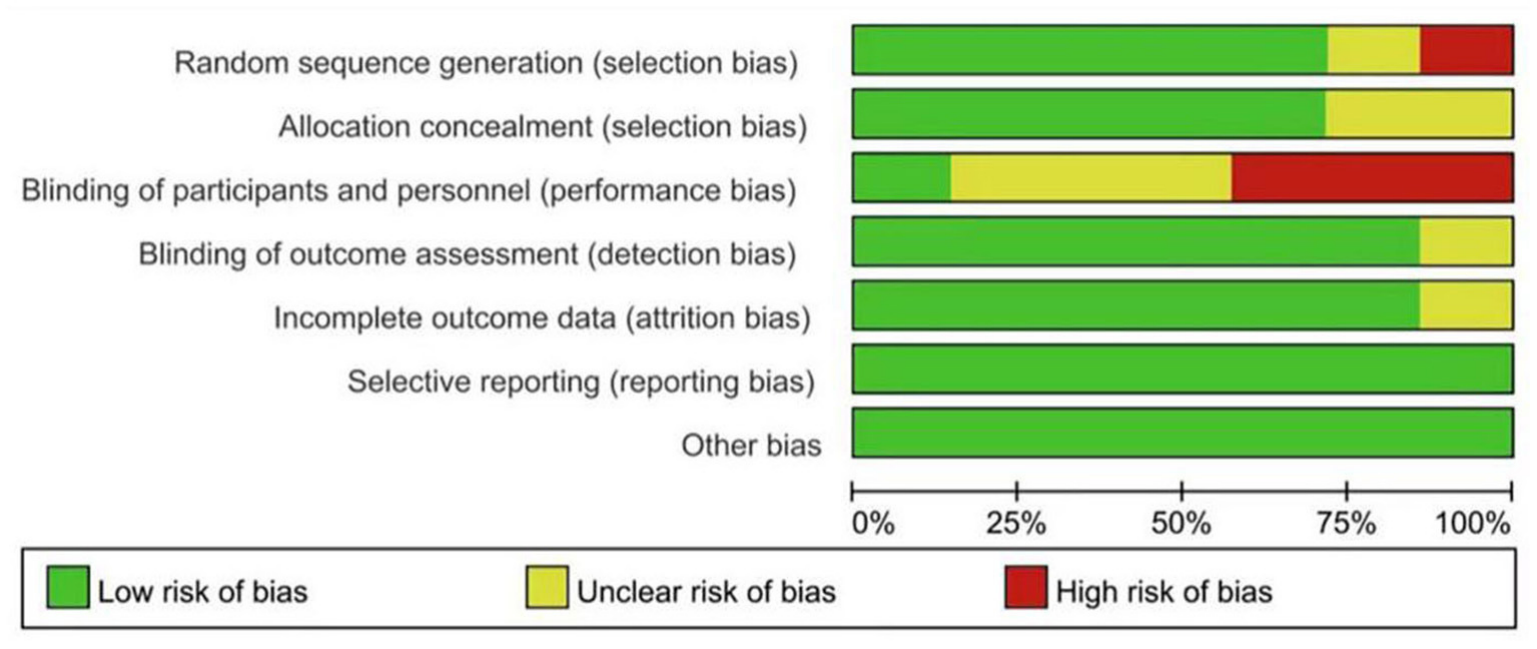
Risk of bias graph of included studies.

**Figure 3:**
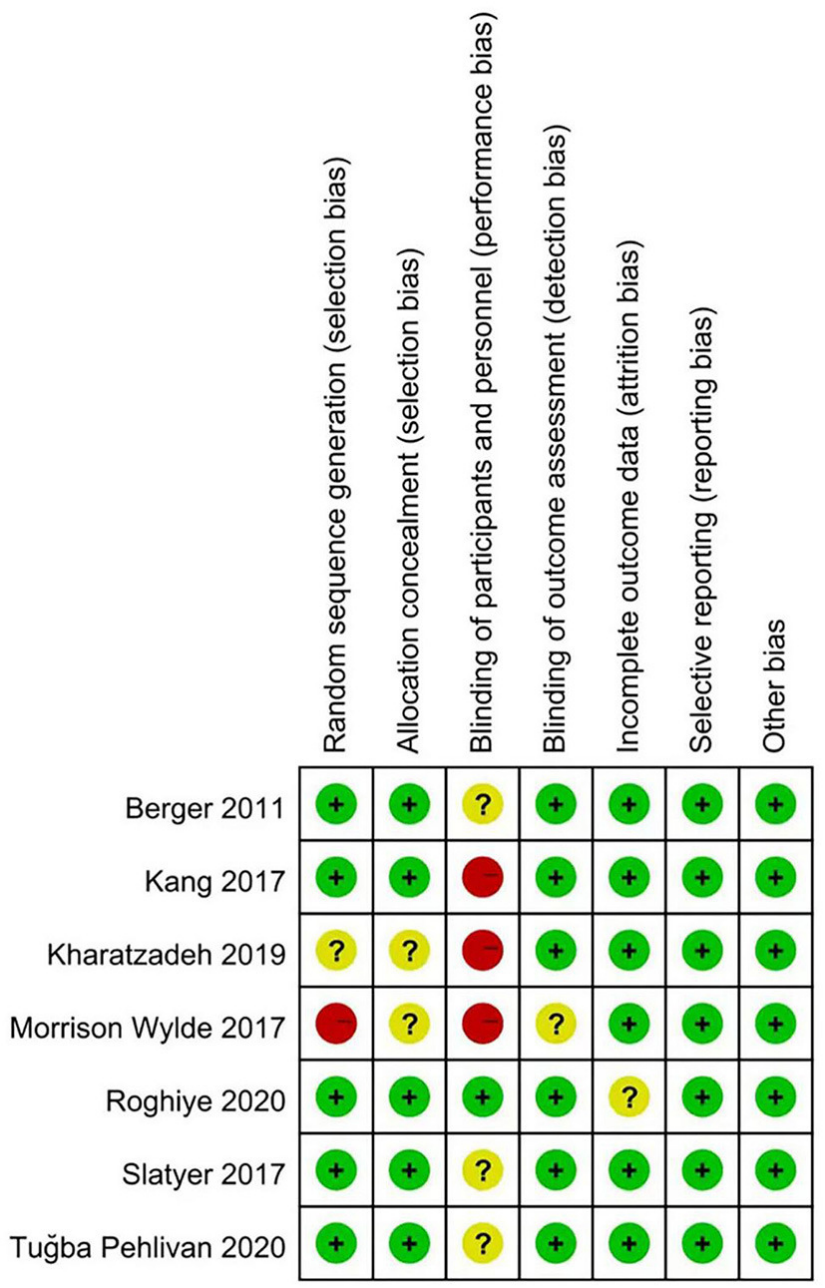
Risk of bias summary of included studies.

Among the included literature, the methodological quality was moderately biased. Two searchers independently conducted a quality assessment for each of the included studies by using the Cochrane Collaboration's risk of bias tool, suggesting that the quality was Grade B. The results of the quality assessment for each study are presented in Figures 2, 3.

### Results of meta-analysis of empathy fatigue, empathy satisfaction and burnout

**Figure 4:**
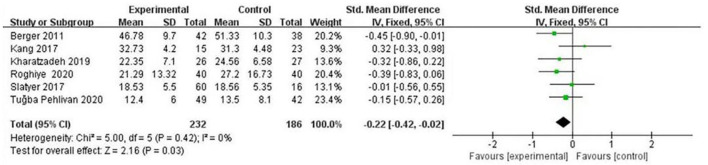
Forest graph showing analysis of empathy fatigue score of nursing staff in psychological intervention group and control group.

**Figure 5:**
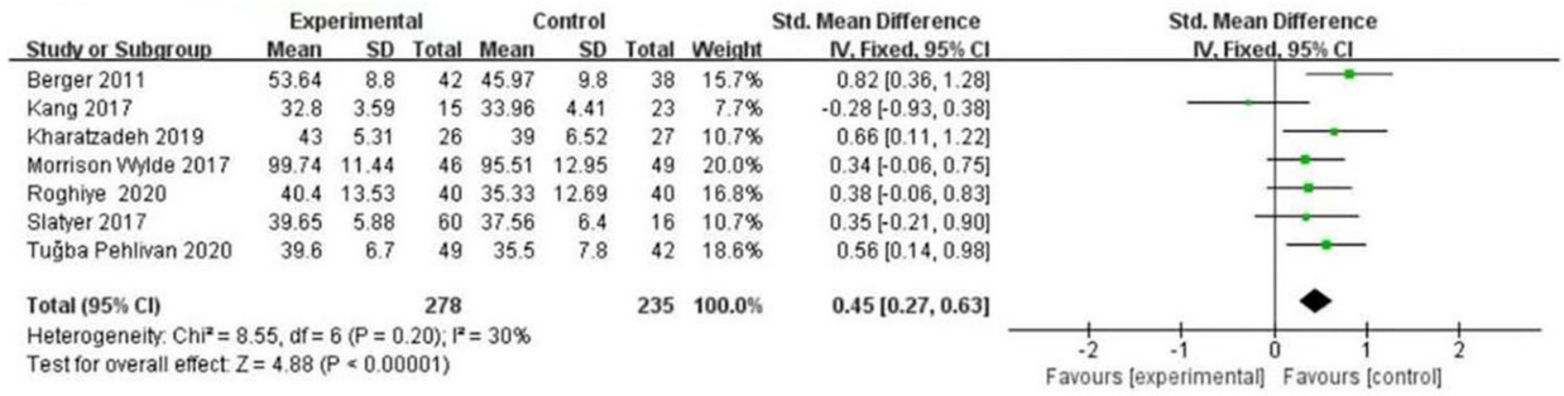
Forest graph showing analysis of empathy satisfaction score of nursing staff in psychological intervention group and control group.

**Figure 6:**
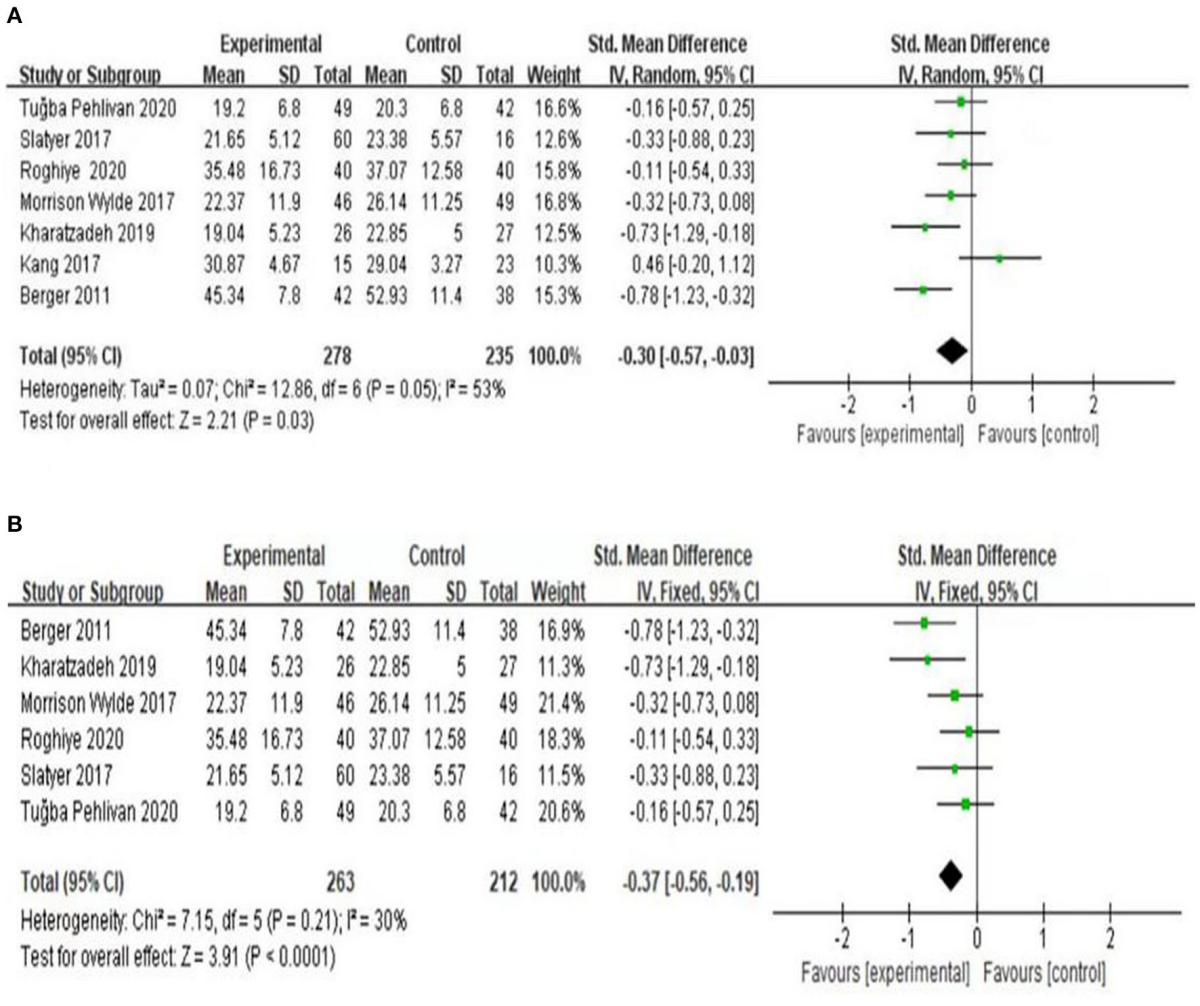
Forest graph showing analysis of burnout score of nursing staff in psychological intervention group and control group. **(A)** A total of seven RCTs were included. **(B)** After changing the criteria of the included studies, Kang's study was excluded for sensitivity analysis.

(1) Effects of psychological intervention on empathy fatigue of nursing staff: a total of six RCTs were included (7, 8, 10–13), covering 418 nurses. The results of the heterogeneity test showed that there was no heterogeneity among the studies (I2 = 0%, *P* > 0.05), so the fixed effects model was chosen. The results showed that the scores of empathy fatigue of nursing staff who accepted psychological intervention were significantly lower than those of the control group. The difference was statistically significant (SMD = ~−0.22, 95% CI: −0.42−0.02, *P* = 0.03) (Figure 4).
(2) Effects of psychological intervention on empathy satisfaction of nursing staff: a total of seven RCTs were included (7–13), involving 513 nurses. The results of the heterogeneity test showed that there was mild heterogeneity among the studies (I2 = 30%, *P* > 0.05), so the fixed effects model was chosen. The results show that the scores of empathy satisfaction of nursing staff who accepted psychological intervention were significantly higher than those of the control group. The difference was statistically significant (SMD = 0.45,95% CI: 0.27~–0.63, *P* < 0.001) (Figure 5).
(3) Effects of psychological intervention on burnout of nursing staff: a total of seven RCTs were included (7–13), covering 513 nurses. The results of the heterogeneity test showed that there was moderate heterogeneity among the studies (I2 = 53%, P = 0.003), so the random effects model was selected. The results suggest that the differences were statistically significant (SMD = −0.30, 95% CI: −0.57−0.03, *P* = 0.03) (A of Figure 6). We changed the criteria of the included studies, that was, the total sample size of each study was not <40. Among the seven included studies, Kang's study (8) had the smallest sample size, with 15 participants in the psychological intervention group and 23 in the control group. The total sample size was 38, therefore, Kang's study was excluded for sensitivity analysis. Moreover, in the sensitivity analysis, we also adopted the method of eliminating studies one by one. When Kang's study was excluded from the analysis, moderate heterogeneity (I2 = 53%, *P* = 0.003) was changed to mild statistical heterogeneity (I2 = 30%, *P* = 0.21), so the fixed effects model was used. The results showed that the burnout scores of the nurses who accepted psychological intervention were lower than those of the control group. The difference was statistically significant (SMD = −0.37, 95% CI: −0.56−0.19, *P* < 0.001), indicating robust results (B of Figure 6). As mentioned above, heterogeneity decreased after Kang's study was excluded. Tracing back to original text, we found that there was no significant difference in the changes of empathy satisfaction and empathy fatigue scores between the experimental group and the control group, indicating that subjects relied on past experience rather than participating in the program immediately after experiencing a child's death. In addition, the objects participated in the project while working continuously, which may also lead to heterogeneity.

### Subgroup analyses

**Figure 7:**
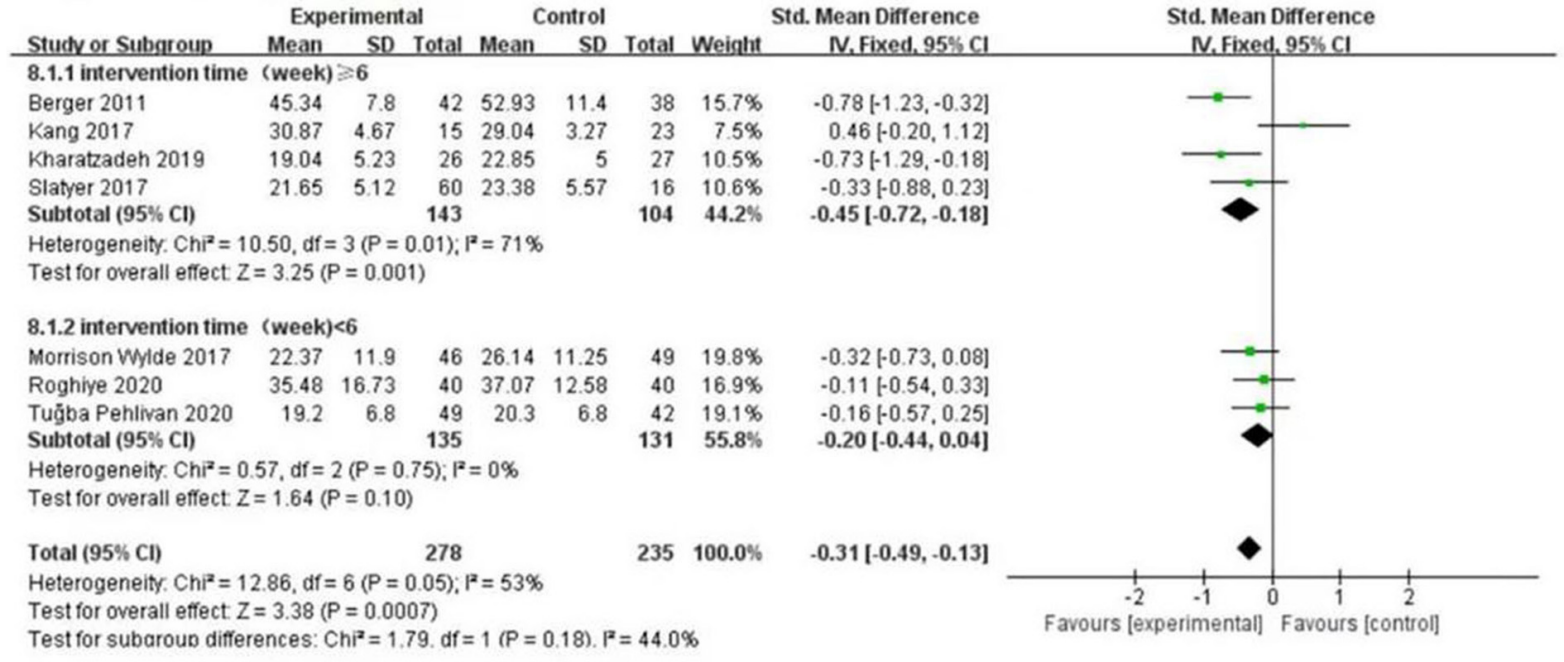
Forest graph showing subgroup analysis of different intervention times.

**Figure 8:**
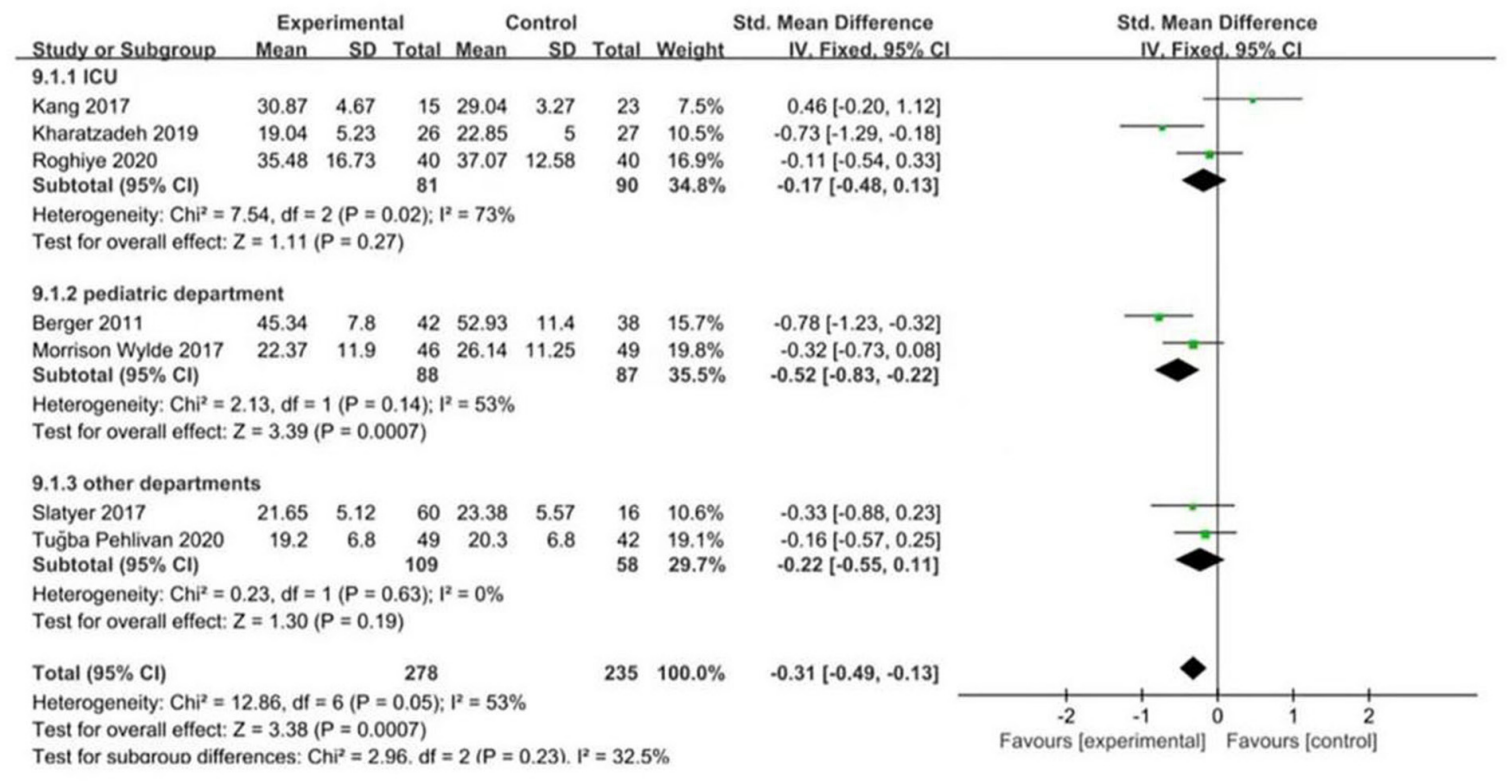
Forest graph showing subgroup analysis of different departments.

According to intervention time and department, the subgroup analysis of seven RCTs (7–13) was carried out in the burnout dimension. First, the nursing staff were divided into two groups on the basis of intervention time. When the intervention time was ≥6 weeks, the result was I2 = 71%, *P* = 0.01; when it was <6 weeks, the result was I2 = 0%, *P* = 0.75. The heterogeneity test results showed that there was moderate heterogeneity among the studies (I2 = 44%, *P* = 0.18). The results are presented in Fig. 7. Likewise, the nursing staff were divided into three groups on the basis of department. The results showed that the ICU department was (I2 = 73%, P = 0.02), pediatric department was (I2 = 53%, P = 0.14) and other departments were (I2 = 0%, *P* = 0.63). The heterogeneity test results showed that there was mild heterogeneity among the included studies (I2 = 32.5%, *P* = 0.23), and the differences were statistically significant (*P* = 0.0007). The results are presented in Figure 8. Therefore, intervention time and department can be considered sources of heterogeneity. From subgroup analysis, the results also showed that when the intervention time was ≥6 weeks, there was statistically significant difference in the level of empathy fatigue between the psychological intervention group and the control group (SMD = −0.45, 95% CI−0.72~−0.18, *P* = 0.001). While <6 weeks, there was no significant difference in the level of empathy fatigue between the psychological intervention group and the control group (SMD = −0.20, 95% CI: −0.44~−0.04, *P* = 0.10). Moreover, for ICU department, there was no significant difference in the level of empathy fatigue between the psychological intervention group and the control group (SMD = −0.17, 95% CI: −0.48~0.13, *P* = 0.27). For pediatric department, there was statistically significant difference in the level of empathy fatigue between the psychological intervention group and the control group (SMD = −0.52, 95% CI: −0.83 to ~0.22, *P* = 0.0007). For other departments, there was statistically significant difference in the level of empathy fatigue between the psychological intervention group and the control group (SMD = −0.22, 95% CI: −0.55~0.11, *P* = 0.19). As showed in Figures 7, 8 subgroup analysis found that the improvement benefit of psychological intervention on burnout of nursing staff has not been determined.

**Figure 9:**
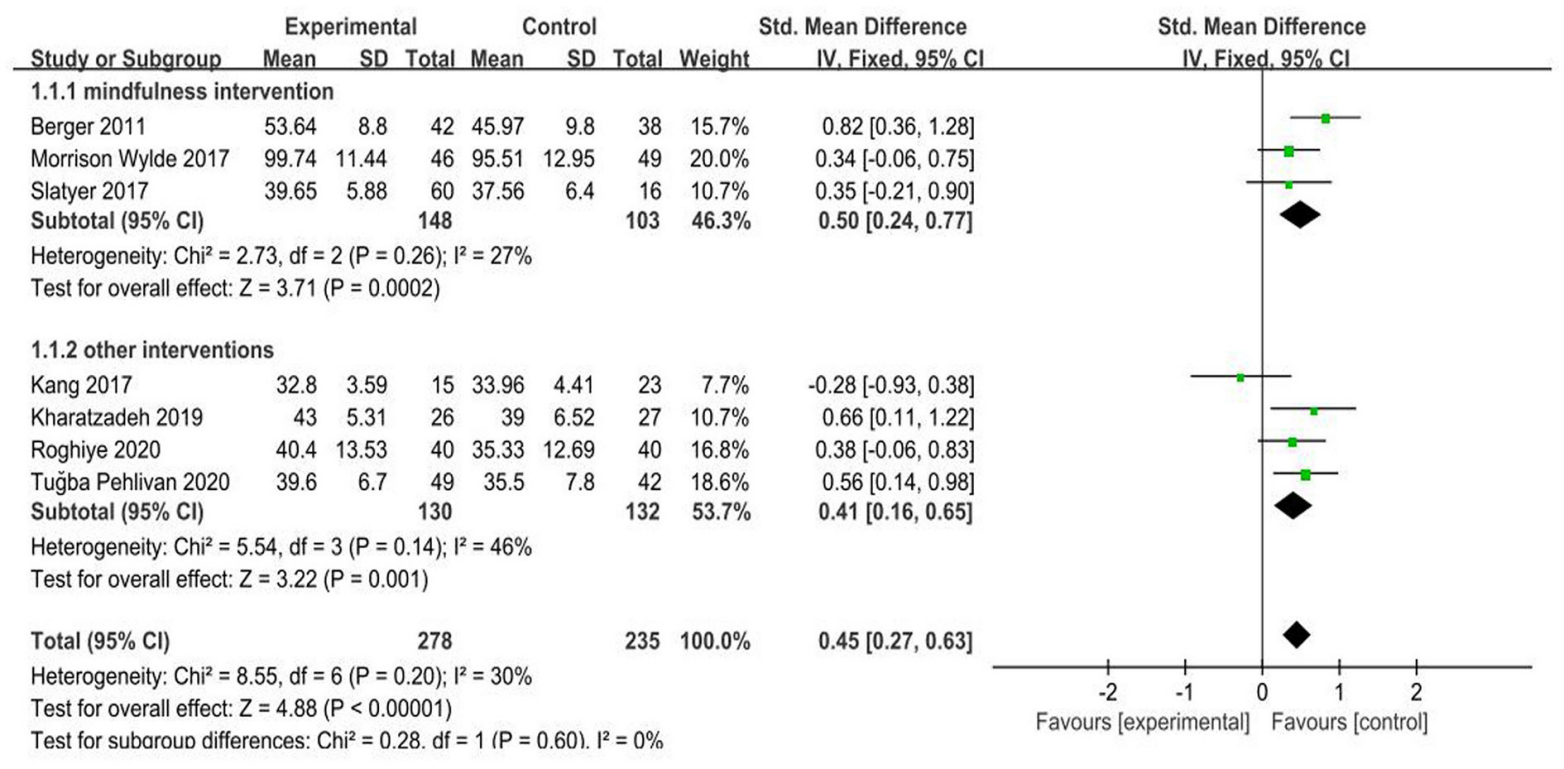
Forest graph showing subgroup analysis of different forms of psychological intervention.

In addition, according to form of psychological intervention, the subgroup analysis of seven RCTs (7–13) was carried out in the empathy satisfaction dimension. From subgroup analysis, the results showed that when using mindfulness intervention, there was statistically significant difference in the level of empathy satisfaction between the psychological intervention group and the control group (SMD = 0.50, 95% CI: 0.24~0.77, *P* = 0.0002). While using other interventions, there was statistically significant difference in the level of empathy satisfaction between the psychological intervention group and the control group (SMD = 0.41, 95% CI: 0.16~0.65, *P* = 0.001) (Figure 9).

### Publication bias

Due to only seven articles being included in the meta-analysis, funnel analysis was unnecessary.

## Discussion

In different areas and departments, empathy fatigue in nurses is very common, and the incidence rate is also very high, which is having a great impact on the nursing profession (14, 15). Zhou's research (16) explored nurse stressors and mediating factors that affect nurses' stress. As for nurse stressors, they basically follow five aspects: nursing profession and work, time distribution and workload, working environment and equipment, patient care and management, and interpersonal relationships. The mediating factors that affect nurses are mainly age, educational background, personality and support system. The quality of the literature included in our study was at a medium level. The methodological quality of the seven articles was moderately biased, and the literature quality was Grade B. Since it was difficult to blind researchers and nursing staff in psychological intervention, this study only included evaluator blindness. Among them, six articles (85.7%) explained specific randomization methods and processes, six articles (85.7%) used outcome evaluator blindness and five articles (71.4%) described the method of allocation scheme hiding. Three articles (42.9%) reported the loss to follow-up of nursing staff, with the rate of loss to follow-up ranging from 0 to 16.3%, and only one explained the reasons. All seven articles all adopted intentionality analysis. All articles compared the baseline data of nursing staff such as age, male-female ratio, working years, marital status, educational background, professional title, empathy fatigue and scores of each dimension, and the results showed that the psychological intervention group and control group were comparable (*P* > 0.05). Therefore, the quality of the literature included in this study was at a medium level, and the research results were relatively reliable. Our results show that the empathy satisfaction score of the psychological intervention group was significantly higher than that of the control group, and the burnout and empathy fatigue scores were significantly lower than that of the control group, indicating that psychological intervention can effectively improve the level of empathy satisfaction of nursing staff and improve the symptoms of burnout and empathy fatigue. The reasons may be as follows: in the course of psychological intervention, nursing staff change their awareness, attention and cognition of people and things through professional theory and technical training. They tap into their own advantages and potential, and enhance their self-regulation ability, enabling them to correctly adjust their inner emotional experience in their work, improve their level of psychological resilience and empathy ability, and effectively alleviate the symptoms of empathy fatigue (8, 9). Empathy fatigue is common among nurses, and the important factors that lead to burnout are mental overload, value conflict and a sense of being out of control (17). Therefore, In order to ensure the sustainable and healthy development of the nursing profession, it is urgent to take necessary psychological intervention measures to improve the condition of empathy fatigue and maintain the mental health of nursing staff.

We found that compared with the control group, psychological intervention can effectively improve the symptoms of empathy fatigue, and the longer the duration of psychological intervention, the better the symptoms of empathy fatigue; in other words, the positive effects of psychological intervention increase with the duration of intervention time, which is similar to the results of previous studies (18). The maximum duration of intervention was 12 weeks in all included literature, among which the immediate effects after intervention were evaluated in all seven articles, and only three followed up after intervention. Therefore, the long-term observation and evaluation of the maintenance effects of psychological intervention are still lacking. At present, there exist innumerable methods of psychological intervention, among which mindfulness therapy and Balint groups are more common for empathy fatigue intervention. The psychological intervention of the empathy fatigue of nursing staff mostly adopts mindfulness therapy, and systematic evaluation shows that this can improve the symptoms of job burnout, anxiety and stress of medical staff, and enhance empathy and concentration (19, 20). Mindfulness therapy, including mindfulness-based stress reduction and cognitive behavioral therapy, is a method to awaken the inner consciousness and observation, and be aware of self-thought, emotions and physical feeling through theoretical learning and training. After more than 30 years of development in foreign countries, the theory has become relatively mature, and the empirical tests and clinical meta-analysis of relevant studies have shown obvious effects (21, 22). The Balint group is a group psychotherapy that integrates psychoanalysis, group therapy, narrative medicine concept, emotional support, self-reflection and so on, which can effectively reduce negative emotions and improve the psychological energy, empathy and mental health level of medical staff (23). The positive affective response to the stimulating situation is mainly affected by cognitive empathy, while the negative affective response is mainly affected by affective empathy (24). The higher the perspective-taking and perspective-selection ability of nurses, the lower the risk of empathy fatigue and job burnout (25). Thus, Balint groups might offer a form of learning to be patient-centered (26) and a method for keeping nurses healthy in their working and living conditions (27). In addition, in terms of other psychological interventions, although there were differences in name, the contents and methods of intervention had some common characteristics through different carriers; for example, popular knowledge manuals, information support materials and videos provided nurses with guidance on empathy fatigue, as well as group therapy, psychological stress management meditation training and so on. It is suggested that nursing managers should provide more targeted empathy fatigue intervention for nursing staff through a variety of forms of psychological intervention (16, 28, 29). According to our results and other research, we suggest regarding psychological intervention courses as a formal training for practicing nurses and new nurse training curricula, enabling more nurses to master these scientific methods of self-decompression management.

In addition, in the context of the COVID-19 global pandemic, the current review found that nurses had higher psychological distress compared to doctors, which was consistent with previous findings that nurses were more vulnerable to stress (30–32). Therefore, that's why we chose nursing staff rather than medical staff. As far as we're concerned, psychological factors are equally effective for medical staff outside of nursing. The COVID-19 pandemic has caused heavy psychological impact among medical workers and the general public. Since most countries around the world are currently prioritizing their medical resources for the containment of COVID-19 and the treatment of patients with COVID-19, there may be limited resources available for psychological services and interventions. Therefore, improving knowledge, awareness, and self-coping strategies are critical in the current situation (33). A prior study has found that 50.4% of study participants had accessed psychological resources through books or media, and 17.5% had sought counseling or psychotherapy. In addition, the study also found that people with mild or lower disturbances preferred to obtain such services from media sources, while those with heavier burdens expressed their needs to seek services directly from professionals (e.g., psychologists, psychiatrists) (34). Empirical evidence suggests mindfulness-based stress reduction (MBSR) programs as an intervention to decrease stress and improve physical and mental health among individuals. Although MBSR studies have demonstrated multiple benefits, the required time commitment impacts clinician participation. Clinicians are less inclined to enroll in programs that require a significant investment of personal time (35). Even so, we do hope that follow-up researches can further explore the psychological problems of medical staff.

### Limitations

(1) the included literature was limited to English, which may generate a certain selection bias; (2) there were many outcome indicators in the included literature, little could be used for combined analysis, and the generalization of the final analysis results was limited; (3) the form, degree and frequency of psychological intervention were different among the included studies, as well as the severity of empathy fatigue, which may affect the authenticity of the results; (4) there is little literature involving different psychological intervention methods, which may affect the accuracy of the results; (5) there was some heterogeneity in the included literature, and the number of studies involved in this meta-analysis was <10, so the funnel plot analysis was not carried out for the time being, and further improvement in meta-analysis methods is demanded in subsequent studies; (6) the sample size included in this study is relatively small, so it is necessary to carry out a large, multi-center, well-designed, high-quality randomized controlled trial for further verification; (7) among the included literature, only two articles performed a secondary trauma score, so this study lacked the dimension analysis of secondary trauma. It is suggested that nursing scholars should perform more studies on the score of the three dimensions of empathy fatigue. Thus, follow-up studies should make updates and additions on this basis.

## Conclusion

Our findings show that psychological intervention can improve the empathy satisfaction level of nurses, improve the symptoms of empathy fatigue and have a certain preventive effect on its occurrence and development. It is suggested that related managers should pay attention to the physical and mental health of nursing staff, and take corresponding measures to improve their level of mental health and quality of nursing, thereby ensuring the sustainable and healthy development of the nursing profession.

## Data availability statement

The original contributions presented in the study are included in the article/supplementary material, further inquiries can be directed to the corresponding author.

## Author contributions

XC, MC, HZ, CW, HC, and ZzZ are the guarantors of the manuscript and take responsibility for the content of this manuscript. XC, ZzZ, RC, and CW contributed to the design of the study. QW, JZ, ZW, and XO were involved in the data analysis. ZhZ, XO, and RC contributed to the acquisition of primary data. XZ, XC, and RC wrote the initial draft of the manuscript and contributed significantly to the revision of the manuscript. All authors read and approved the final manuscript.

## Funding

This study was funded by the Natural Science Foundation of Guangdong Province (2021A1515011373) and Science and Technology Development Special Fund Competitive Allocation Project of Zhanjiang (2021A05108).

## Conflict of interest

The authors declare that the research was conducted in the absence of any commercial or financial relationships that could be construed as a potential conflict of interest. The reviewer QT declared a shared parent affiliation with the authors HL, JL, XO, ZhZ, ZW, and RC to the handling editor at the time of review.

## Publisher's note

All claims expressed in this article are solely those of the authors and do not necessarily represent those of their affiliated organizations, or those of the publisher, the editors and the reviewers. Any product that may be evaluated in this article, or claim that may be made by its manufacturer, is not guaranteed or endorsed by the publisher.
